# Data to assess the mediation effect of perceived responsibility for environmental damage on the relationship between moral identity and green consumption

**DOI:** 10.1016/j.dib.2018.09.101

**Published:** 2018-10-02

**Authors:** Bo Wu, Zhiyong Yang

**Affiliations:** a Business School, Tianjin University of Finance & Economics, Tianjin, China; b Bryan School of Business and Economics, The University of North Carolina Greensboro, United States

## Abstract

This Data in Brief article is for Study 3 in the manuscript # JEVP-2018-140 (“The Impact of Moral Identity on Consumers׳ Green Consumption Tendency: The Role of Perceived Responsibility for Environmental Damage”). It examines whether responsibility for environmental damage mediates the relationship between moral identity and green consumption. The data was collected using lab experiment. Participants were randomly assigned to moral-identity-activated condition and moral-identity-not-activated condition. The choice of eco-friendly product relative to conventional counterpart was measured. Responsibility for environmental damage was measured through a six-item scale. 65 American undergraduate students took part in the experiment. Data was analyzed employing SPSS. Logistic regression and mediation analysis were used as statistical tool of analysis.

**Specifications table**

**Table:** 

Subject area	Psychology
More specific subject area	Consumer behavior, green consumption, moral identity
Type of data	Table
How data was acquired	Experiment
Data format	Raw
Experimental factors	Sample consisted of undergraduate students from a major university in the U.S.
Experimental features	Moral identity is manipulated; Green consumption is measured through eco-friendly product choice relative to conventional counterpart; Responsibility for environmental damage is measured through a six-item scale
Data source location	United States
Data accessibility	Data is included in this article

**Value of the data**

● This data provides information on eco-friendly product choice relative to conventional counterpart and responsibility for environmental damage across moral-identity-activated condition and moral-identity-not-activated condition using American sample.
● The effect of moral identity on green consumption can be compared with the effect of moral identity on other prosocial behavior.
● The effect of moral identity on green consumption can be compared with other cultures or countries.
● The mediation effect of responsibility for environmental damage can be compared with other mediation effect.

## 1 Data

The data comprised experiment data on eco-friendly product choice relative to conventional counterpart and responsibility for environmental damage across moral-identity-activated condition and moral-identity-not-activated condition, manipulation checks and demographics. Moral identity refers to a structured cognitive schema of moral values, goals, traits and behavioral scripts [1]. It was manipulated using the handwriting task. After writing their stories, participants completed a manipulation check for moral identity, using the question “*How much does the story you wrote in the handwriting task reflect how you see yourself as: (1) a student, (2) a member of an organization, (3) a moral person, and (4) safety conscious*?” (1 = *not at all*; 7 = *very much so*). Perceived responsibility for environmental damage was measured through a six-item scale adapted from previous research [2]. Green consumption refers to the extent to which consumers consider the impact of their own behavior on the environment when they purchase, use, or dispose of products, and try to minimize the negative impact and maximize the positive impact on the environment [3]. Green consumption was measured using eco-friendly notebook choice relative to conventional notebook. Participants were asked to make a choice between notebook Option 1 and Option 2. Option 1 was with more sheets and larger, while Option 2 was more eco-friendly. Finally, participants responded to demographic questions, including gender and age.

Manipulation checks reveal that compared with participants in the moral-identity-not-activated condition (*M* = 4.47), those in the moral-identity-activated condition indicated that their stories were more reflective of how they saw themselves as moral people (*M* = 5.82; *t* (48) = 3.26, *p* < .01). However, the extent to which participants saw themselves as students, members of an organization, or safety conscious did not differ across moral identity activated and non-activated conditions (*p*׳s > .45).

A logistic regression on the choice of Option 2 (the eco-friendly notebook), using moral identity as the independent variable, and demographics as covariates, revealed significant effects of moral identity (*b* = 1.54, Wald *χ*^2^ = 7.51, *p* = .006). Consistent with our expectations, a greater percentage of people in the moral-identity-activated group (66.67%) chose Option 2, compared to 34.38% in the moral-identity-not-activated group. Gender and age had no effect on the notebook choice (*p*׳s > .11).

To test whether the relationship between moral identity and green consumption was mediated by perceived responsibility for environmental damage, a mediation analysis using the PROCESS macro (Model 4, *n* = 5000; [4]) with moral identity as the independent variable, responsibility for environmental damage as the mediator, and gender and age as covariates was conducted. The results indicated that the indirect effect of moral identity on the choice of eco-friendly notebook was positive (1.12) and significant (95% CI, .21 to 2.68, excluded zero).

## 2 Experimental design, materials and methods

The data presented a quantitative research based on an experiment design to assess the effect of moral identity on green consumption and the mediation effect of responsibility for environmental damage [5]. Experiment method was deemed suitable for data collection. Sixty-five undergraduate students (25 females; M age = 23.95, SD = 3.93) from a major university in the U.S. participated in the study in exchange for a paper notebook.

A handwriting task was used to manipulate the salience of moral identity (activated vs. not). Participants received nine words, and wrote one or a few stories about themselves that used each word at least once. In the moral-identity-activated condition, the nine words were the traits that people commonly associate with being a moral person (e.g., caring, fair, and kind; [6]). To manipulate moral identity more strictly, in the moral-identity-not-activated condition, the nine words were positive traits unrelated to morality (e.g., polite, and happy). This kind of manipulation has also been well used in the literature [7], [8]. After writing their stories, participants completed a manipulation check, using the question “How much does the story you wrote in the handwriting task reflect how you see yourself as: (1) a student, (2) a member of an organization, (3) a moral person, and (4) safety conscious?” (1 = not at all; 7 = very much so). Subsequently, a six-item measure of perceived responsibility for environmental damage adapted from previous research [2], and demographics (gender and age) were measured.

After completing these two tasks, participants took the notebook of their choice as compensation for their participation. They chose from two options with the same price: Option 1 (Five Star notebook) was a conventional notebook with 100 sheets at the size of 11 × 9.8 × 0.8 in., whereas Option 2 (TOPS second nature notebook) was an eco-friendly notebook with 80 sheets at the size of 9.5 × 6 × 0.2 in. The data was coded and inputted in SPSS version 25. Data was analyzed through logistic regression analysis.

## References

[1] Choi W.J., Winterich K.P. (2013). Can brands move in from the outside? How moral identity enhances out-group brand attitudes. J. Mark..

[2] Peloza J., White K., Shang J. (2013). Good and guilt-free: the role of self-accountability in influencing preferences for products with ethical attributes. J. Mark..

[3] Carlson L., Grove S.J., Kangun N. (1993). A content analysis of environmental advertising claims: a matrix method approach. J. Advert..

[4] Hayes A.F. (2013). Introduction to Mediation, Moderation, and Conditional Process Analysis: A Regression-based Approach.

[5] Wu B., Yang Z. (2018). The impact of moral identity on consumers’ green consumption tendency: The role of perceived responsibility for environmental damage. J. Environ. Psychol..

[6] Aquino K., Reed II A. (2002). The self-importance of moral identity. J. Pers. Soc. Psychol..

[7] Aquino K., Reed II A., Thau S., Freeman D. (2007). A grotesque and dark beauty: how moral identity and mechanisms of moral disengagement influence cognitive and emotional reactions to war. J. Exp. Soc. Psychol..

[8] Reed II A., Kay A., Finnel S., Aquino K., Levy E. (2016). I don’t want the money, I just want your time: how moral identity overcomes the aversion to giving time to prosocial causes. J. Pers. Soc. Psychol..

